# Spatial spread of COVID-19 during the early pandemic phase in Italy

**DOI:** 10.1186/s12879-024-09343-8

**Published:** 2024-04-29

**Authors:** Valeria d’Andrea, Filippo Trentini, Valentina Marziano, Agnese Zardini, Mattia Manica, Giorgio Guzzetta, Marco Ajelli, Daniele Petrone, Martina Del Manso, Chiara Sacco, Xanthi Andrianou, Antonino Bella, Flavia Riccardo, Patrizio Pezzotti, Piero Poletti, Stefano Merler

**Affiliations:** 1 Center for Health Emergencies, Fondazione Bruno Kessler, Trento, Italy; 2 Department of Physics and Astronomy “Galileo Galilei”, University of Padua, Padua, Italy; 3 Dondena Centre for Research On Social Dynamics and Public Policy, Bocconi University, Milan, Italy; 4 Department of Decision Sciences, Bocconi University, Milan, Italy; 5 Laboratory for Computational Epidemiology and Public Health, Department of Epidemiology and Biostatistics, Indiana University School of Public Health, Bloomington, IN USA; 6 Department of Infectious Diseases, Istituto Superiore Di Sanità, Rome, Italy; 7 Department of Statistics, Sapienza University of Rome, Rome, Italy

**Keywords:** COVID-19, SARS-CoV-2, Transmission distance, Infection spread, Spatial diffusion, Spatial model

## Abstract

Quantifying the potential spatial spread of an infectious pathogen is key to defining effective containment and control strategies. The aim of this study is to estimate the risk of SARS-CoV-2 transmission at different distances in Italy before the first regional lockdown was imposed, identifying important sources of national spreading. To do this, we leverage on a probabilistic model applied to daily symptomatic cases retrospectively ascertained in each Italian municipality with symptom onset between January 28 and March 7, 2020. Results are validated using a multi-patch dynamic transmission model reproducing the spatiotemporal distribution of identified cases. Our results show that the contribution of short-distance ($\le 10 km)$ transmission increased from less than 40% in the last week of January to more than 80% in the first week of March 2020. On March 7, 2020, that is the day before the first regional lockdown was imposed, more than 200 local transmission foci were contributing to the spread of SARS-CoV-2 in Italy. At the time, isolation measures imposed only on municipalities with at least ten ascertained cases would have left uncontrolled more than 75% of spillover transmission from the already affected municipalities. In early March, national-wide restrictions were required to curb short-distance transmission of SARS-CoV-2 in Italy.

**Supplementary Information:**

The online version contains supplementary material available at 10.1186/s12879-024-09343-8.

## Introduction

The assessment of the potential spatial spread of an infectious pathogen in human populations is key to design interventions to effectively contain local outbreaks, control the ongoing transmission, and avoid widespread epidemics.

The sudden increase in ascertained cases of COVID-19 worldwide experienced in 2020 [1–3] led several countries to apply unprecedented restrictions at the national level, including national lockdowns, to mitigate the pressure caused by the increasing number of patients on healthcare systems [2, 4]. Difficulties in implementing effective containment measures in early 2020 were determined by a combination of factors, including the high proportion of silent transmission caused by asymptomatic cases, the initial lack of knowledge about SARS-CoV-2 epidemiology, and the limited availability of diagnostic tests during the first months of the pandemic [5].

During the initial phase of an epidemic, sporadic importation of cases from most affected areas often results in geographical heterogeneities in the infection spread [6–8]. In this phase, containment measures should rely on the timely detection of cases, but the definition of the adequate spatial scale of control strategies would benefit from the identification of areas representing important sources of national spreading [9–11]. Whether adopting local restrictions to contain the spatial spread of a new emerging pathogen is more appropriate than moving towards national-level interventions depends on a variety of factors. These include the distance at which the infection transmission likely occurs and how the number of local clusters of cases could increase over time and across different geographical areas in the absence of restrictions.

Several models proposed to describe the spatiotemporal spread of epidemics integrate data quantitatively describing human mobility patterns, e.g. the number of individuals commuting or traveling across different areas as estimated from surveys or census data [12–14] or detailed records collected through mobile phones and wearable devices to measure individuals' movements over different periods of time [15]. A wide set of models of human mobility have been also developed to describe movement fluxes between areas in terms of population densities and distance [16]. The underlying assumption of most of these approaches is that population flows are driving the pathogen spread among the individuals. However, a quantitative assessment of the risk of SARS-CoV-2 transmission at different distances from epidemiological data is still lacking.

On February 20, 2020, Italy was the first country where a locally acquired infection of SARS-CoV-2, with no link to cases directly or indirectly related to endemic areas, was identified in Europe [2]. Several studies have already highlighted that COVID-19 was already circulating in the country well before this date [2, 17, 18] with possible marked heterogeneity in the epidemic risk across different regions [19].

The aim of our work is to leverage consolidated and detailed records of COVID-19 symptomatic cases occurring in Italy in early 2020 to investigate the transmission of SARS-CoV-2 within and between municipalities, quantifying the proportion of local transmission and the number of transmission foci likely present in the country before a lockdown was imposed in Italy. We here analyze the time series of cases by symptom onset between January 26 to March 7, 2020 (corresponding to the 6 epidemiological weeks preceding the first regional lockdown in Italy) as obtained for each municipality of Italy by retrospective investigations of cases and case contacts [2]. By using a probabilistic approach, we investigate the likely source locations of infection of COVID-19 cases in the early pandemic phase and we estimate the contribution of each municipality to the spread of SARS-CoV-2 both locally and at the national level.

## Methods

### Study population and data

The first autochthonous case of COVID-19 in Italy was microbiologically diagnosed in the Lombardy Region on February 20, 2020. At the time, intensive testing, isolation of confirmed cases, and quarantine of case contacts were in place in the entire country [19]. Following the rapid increase of SARS-CoV-2 laboratory-confirmed infections, local and national health authorities imposed increasingly strict physical distancing measures, with a quarantine imposed on all individuals residing in 10 municipalities in the Lombardy Region and one in the Veneto Region on February 23, 2020 [2]. A regional lockdown in Lombardy and a national lockdown were imposed respectively on March 8 and March 10, 2020 [20]. Applied measures included the suspension of teaching activities and restrictions on individuals’ movements across different regions and culminated in the closure of all non-essential retail and shops and a “stay at home” order applied throughout the entire Italian territory.

Since January 2020, data on PCR-confirmed SARS-CoV-2 infections have been collected in the 19 Italian Regions and the two Autonomous Provinces and reported to National Integrated Surveillance System [19]. A central database of all infections confirmed in Italy was formally established the February 27, 2020 and managed by the Italian National Institute of Health. For any confirmed infection, information was collected on the date of diagnosis, municipality of residence, and clinical severity; the date of symptom onset was also recorded for symptomatic cases. The initial line list of laboratory-confirmed cases was retrospectively consolidated, through information gathered with standardized interviews to ascertained infections and PCR testing of their close contacts.

Our analysis is based on the consolidated dataset of all ascertained cases with symptom onset between January 26 and March 7, 2020, corresponding to the 6 epidemiological weeks preceding the first regional lockdown imposed in Lombardy on March 8, 2020. We focus our analysis on this period to reduce the potential biases led by the introduction of strict restrictions to the population. Data used to perform the presented analysis were extracted in February 2021.

### Probabilistic approach

By adapting a method previously developed to estimate sources and sinks of malaria parasites in Madagascar [21], we investigate the likely source locations of infection of each symptomatic case retrospectively identified by public health authorities in Italy with symptom onset in the 6 weeks between January 26 and March 7. For each case residing in municipality *i* with symptom onset on day *t*, we describe the risk that the case was infected *T* days previously because of contacts with people residing in the municipality *j* as:

$${{\text{L}}}_{{\text{i}},{\text{j}}}\left(t,T\right)={C}_{i,j}\mathcal{G}\left(T\right)\frac{{Y}_{j}\left(t-T\right)}{{N}_{j}}$$

where ${C}_{i,j}$ represents the number of individuals daily traveling from $i$ to $j$, $\mathcal{G}\left(T\right)$ is the probability distribution of the SARS-CoV-2 generation time (assumed to be equal to the distribution of the serial interval estimated in [2]), ${Y}_{j}\left(t-T\right)$ is the number of infected individuals residing in *j* who developed symptoms at time $t-T$, and ${N}_{j}$ is the total number of individuals residing in *j.*

The amount of travels across the different municipalities of Italy (${C}_{i,j}$) is modeled by means of a radiation model [22], which is based on data on the size of the population residing in each municipality, the distance between their centroids, and the proportion of daily commuters recorded by Italian National Institute of Statistics in 2019 (Figure S1) [23].

We estimate the probability that a case residing in municipality *i* with symptom onset on day *t*, was infected by a case residing in municipality *j* as:

$${{\text{p}}}_{{\text{i}},{\text{j}}}\left(t\right)=\frac{{\sum }_{T=1}^{\infty }{L}_{i,j}\left(t,T\right)}{{\sum }_{j=1}^{M}{\sum }_{T=1}^{\infty }{L}_{i,j}\left(t,T\right)}$$

where *M* is the total number of municipalities in Italy in 2020 (namely, 7926).

Similarly, the probability that a case residing in municipality *i* and developing symptoms during the period $\uppi$ was infected by a case from municipality *j* is computed as:

$${{\text{p}}}_{{\text{i}},{\text{j}}}\left(\uppi \right)=\frac{{\sum }_{t\in\uppi }{p}_{i,j}\left(t\right){{\text{Y}}}_{{\text{i}}}\left({\text{t}}\right)}{{\sum }_{t\in\uppi }{{\text{Y}}}_{{\text{i}}}\left({\text{t}}\right)}.$$

Finally, we estimate the probability that individuals developing symptoms during the period $\uppi$ were infected within a distance *D* from their residence as:

$${p}_{D}\left(\uppi \right)=\frac{{\sum }_{i}{\sum }_{j:{d}_{i,j}<D}{p}_{i,j}\left(\uppi \right){Y}_{i}\left(\uppi \right)}{{\sum }_{i}{Y}_{i}\left(\uppi \right)}$$

where possible sources *j* run over all municipalities with a distance from *i (*namely, ${d}_{i,j}$) lower than *D*.

The contribution of each municipality *j* in the number of infection episodes occurring at time $t$ in all the other municipalities of Italy is quantified as ${\sum }_{i\ne j}{p}_{i,j}\left(t\right){Y}_{i}\left(t\right)/{\sum }_{{\text{j}}=1}^{{\text{M}}}{\sum }_{i\ne j}{p}_{i,j}\left(t\right){Y}_{i}\left(t\right)$.

We estimate the number of epidemic foci occurred in Italy up to March 7, 2020. To this aim, we identify for each week $w$ those municipalities characterized by a non-negligible number of ascertained symptomatic cases (${\sum }_{t\in w}{{\text{Y}}}_{{\text{i}}}\left({\text{t}}\right)>10$) and incidence (${\sum }_{t\in w}{{\text{Y}}}_{{\text{i}}}\left({\text{t}}\right)/{{\text{N}}}_{{\text{i}}}>0.001$), and by the majority of transmission episodes estimated as occurring between individuals residing in the municipality (${p}_{i,i}\left(w\right)>0.5$).

### Mobility patterns and model validation through a dynamic transmission model

In the probabilistic approach, we assume that the mobility fluxes among municipalities can be modeled through a radiation model. Although the radiation model has been effectively employed to describe the spatial spread of infectious diseases in high-income countries [22, 24], following the approach already used in Gatto et al. [13], we show that the flows of individuals obtained through the radiation model are in good agreement with mobility data across the 12 provinces of the Lombardy region, based on 2016 census data adjusted with the population projections for 2020 [25] (see Figures S2 and S3). Furthermore, we use a dynamic metapopulation transmission model based on a susceptible-infectious-recovered (SIR) schema to test if the radiation model is reasonably able to capture the observed spatial spread of COVID-19 in Italy and the overall temporal increase of COVID-19 patients across regions from February 1 up to March 7, 2020. To compare model simulations with data, we assume that 3% of all infections were ascertained by public health authorities, either in real time or retrospectively through contact tracing operations and epidemiological investigations [26]. In the dynamic model, infected individuals residing in the municipality *j* are assumed to exert a time dependent force of infection ${\lambda }_{i,j}\left(t\right)$ on individuals residing in municipality $i$ defined as ${\lambda }_{i,j}\left(t\right)=\beta {C}_{i,j}{I}_{j}\left(t\right)/{{\text{N}}}_{j}$, where $\beta$ is the SARS-CoV-2 transmission rate, ${C}_{i,j}$ is the amount of individuals daily traveling from $i$ to $j$ as obtained by using the radiation model, ${I}_{j}(t)$ and ${N}_{j}$ are, respectively, the overall number of infectious individuals and the population size in municipality $j$. Based on the simulation results, we compute the probability that an individual residing in municipality *i* and infected at day *t* was infected by a case from municipality *j* as ${{\text{p}}}_{{\text{i}},{\text{j}}}\left(t\right)={\uplambda }_{i,j}\left(t\right)/{\sum }_{j=1}^{M}{\lambda }_{i,j}\left(t\right)$, with *M* representing the overall number of municipalities of Italy in 2020; ${{\text{p}}}_{{\text{i}},{\text{j}}}\left(\uppi \right)$ is computed as in the probabilistic approach, but using the overall number of infections estimated by the dynamic model instead of the symptomatic cases ascertained in the data. Given the large uncertainty surrounding the ability of the public health system in identifying (either in real time or retrospectively) cases that occurred in the early pandemic phase, we repeat the analysis and estimate the risk of SARS-CoV-2 transmission at different distances by assuming also a 10% ascertainment ratio.

The SIR model is parametrized to reproduce at the national level an epidemic curve associated with an exponential growth rate $r$ corresponding to a basic reproduction number ${R}_{0}=2.8$, representing the transmissibility potential of SARS-CoV-2, estimated for the Lombardy Region between February 12 and March 9, 2020 [2, 20]. The average duration of the infectivity period is assumed to be equal to the mean serial interval $G$ [2]. The ${R}_{0}$ associated with the simulated epidemic curve is computed by considering the growth rate $r$ associated with the number of new cases simulated by the model at the national level and using the standard equation ${R}_{0}=1+rG$. The model is initialized on February 1 (at ${t}_{0}=0$) with a number of infected individuals ${I}_{0}$ that is consistent with the ascertainment ratio in Italy during the early pandemic phase (3% by March 8, 2020 [26]; 10% was considered for sensitivity analysis), and the consolidated number of ascertained cases developing symptoms before strict restrictions were imposed on the general population (namely, 517 individuals on February 23, 2020). The dynamic model considered in this work is deterministic. However, initial infections are distributed over the national territory by random sampling from a multinomial distribution with probabilities proportional to the cumulative number of symptomatic cases retrospectively identified in Italy across the different municipalities as of February 15, 2020. To explore the uncertainty characterizing the initial spatial dispersal of SARS-CoV-2 infections, model simulations are repeated 100 times by randomly sampling the municipalities of residence of infectious individuals at the start of simulations. Results are presented both in terms of model mean estimates and 95% Prediction Intervals (PI) associated with different initial conditions, and in terms of model estimates associated with initial conditions minimizing the root mean square error between the time series of cases retrospectively identified at the regional level and those estimated by simulating the dynamic SIR model.

## Results

### Spatial dispersal and transmission foci of SARS-COV-2

The retrospective and prospective investigation of the first laboratory-confirmed cases identified 1,274 COVID-19 cases with symptom onset between January 28 and February 20 (i.e., the earliest date of diagnosis of an autochthonous case in Italy), already scattered across more than 600 municipalities in 18 regions. Between February 20 and March 7, public health authorities confirmed 8,596 COVID-19 cases (Table 1), while additional 15,402 symptomatic cases with symptom onset in this period were retrospectively ascertained in the following weeks [2]. According to the consolidated dataset, as of March 7, 2020, new COVID-19 cases were emerging in 2,644 municipalities and all regions were affected by sustained SARS-CoV-2 transmission (Table 2, Figure S4).

**Table 1:** Identified symptomatic cases by date of notification across the six epidemiological weeks between January 26 and March 7, 2020

	Identified cases by date of notification		
Week	Number of cases	Number of municipalities *(% of the total 7,926)*	Number of regions *(% of the total 20)*
Jan 26 – Feb 01	0	0 *(0%)*	0 *(0%)*
Feb 02 – Feb 08	0	0 *(0%)*	0 *(0%)*
Feb 09 – Feb 15	0	0 *(0%)*	0 *(0%)*
Feb 16 – Feb 22	88	42 *(0.5%)*	4 *(20%)*
Feb 23 – Feb 29	1,540	533 *(6.7%)*	16 *(80%)*
Mar 01 – Mar 07	6,968	1,490 *(18.8%)*	20 *(100%)*

**Table 2:** Retrospectively identified symptomatic cases by date of symptom onset across the six epidemiological weeks between January 26 and March 7, 2020

	Retrospectively identified cases by date of symptoms onset		
Week	Number of cases	Number of municipalities *(% of the total 7,926)*	Number of regions *(% of the total 20)*
Jan 26 – Feb 01	168	144 *(1.8%)*	15 *(75%)*
Feb 02 – Feb 08	174	141 *(1.8%)*	10 *(50%)*
Feb 09 – Feb 15	413	271 *(3.4%)*	14 *(70%)*
Feb 16 – Feb 22	1,606	608 *(7.7%)*	18 *(90%)*
Feb 23 – Feb 29	5,822	1,408 *(17.8%)*	20 *(100%)*
Mar 01 – Mar 07	17,089	2,644 *(33.3%)*	20 *(100%)*

**Fig. 1:**
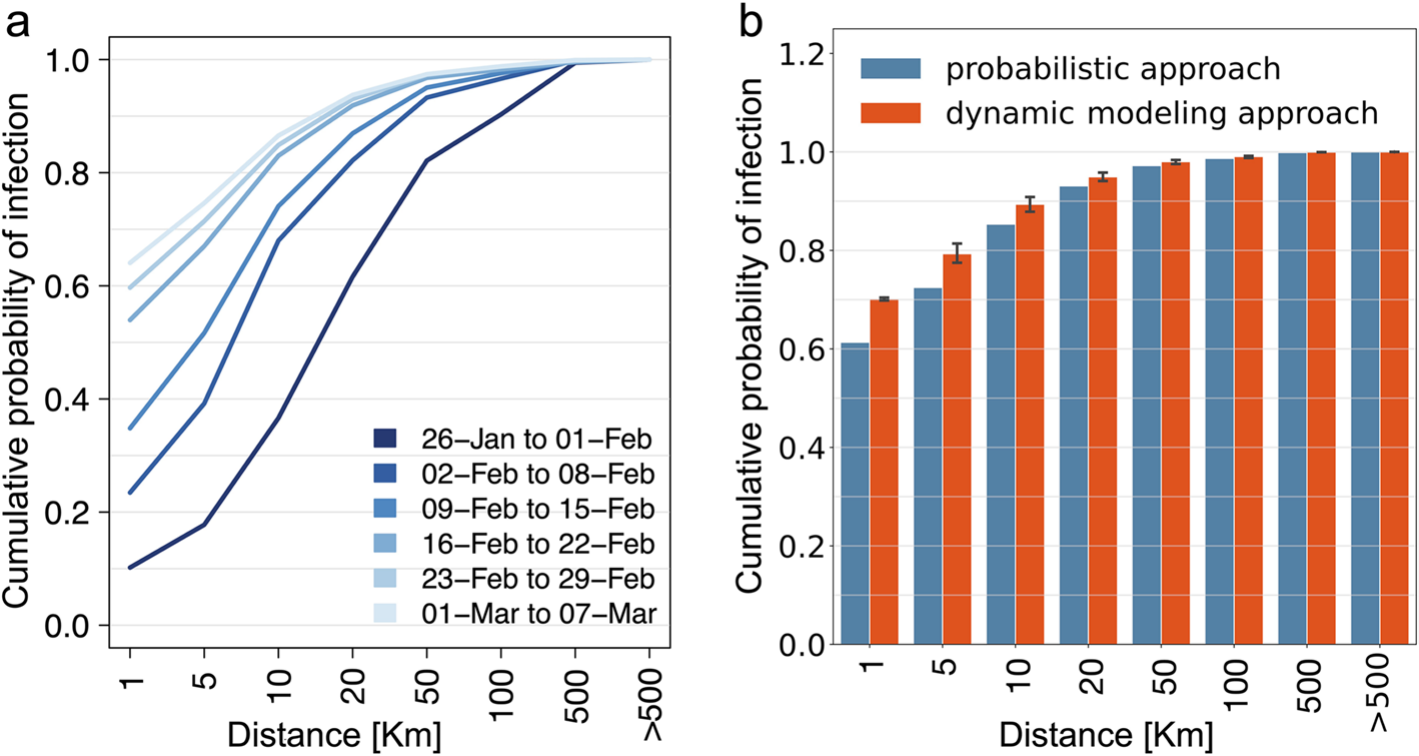
**a** Cumulative distribution of the probability that a COVID-19 case was infected at a distance *D* from their residence, as estimated with the probabilistic approach, for 6 consecutive weeks between January 26 and March 7, 2020. **b** Cumulative distribution of the probability that a COVID-19 case was infected at distance *D* from their residence, as estimated with the probabilistic approach (blue) and the dynamic SIR model (red), considering the entire time interval between January 26 and March 7, 2020. Vertical lines show the range from 2.5 to 97.5 percentiles associated with 100 simulation runs

Our analysis shows that up to February 1, 2020, less than 20% of transmission episodes occurred because of interactions between individuals residing 5 km apart (Fig. 1a), with more than 20% of cases likely infected at more than 50 km from their municipality of residence. In this period, less than 40% of transmission episodes were likely occurring at a distance within 10 km (see Figure S5). In contrast, we estimate that after mid-February more than 80% of transmission events were occurring within 10 km, while less than 5% of cases were attributable to transmission episodes occurring at a distance greater than 20 km (Fig. 1a). Estimates of the overall cumulative distribution of the geographic distances of transmission that occurred up to March 7, 2020, can be found in Fig. 1b.

**Fig. 2:**
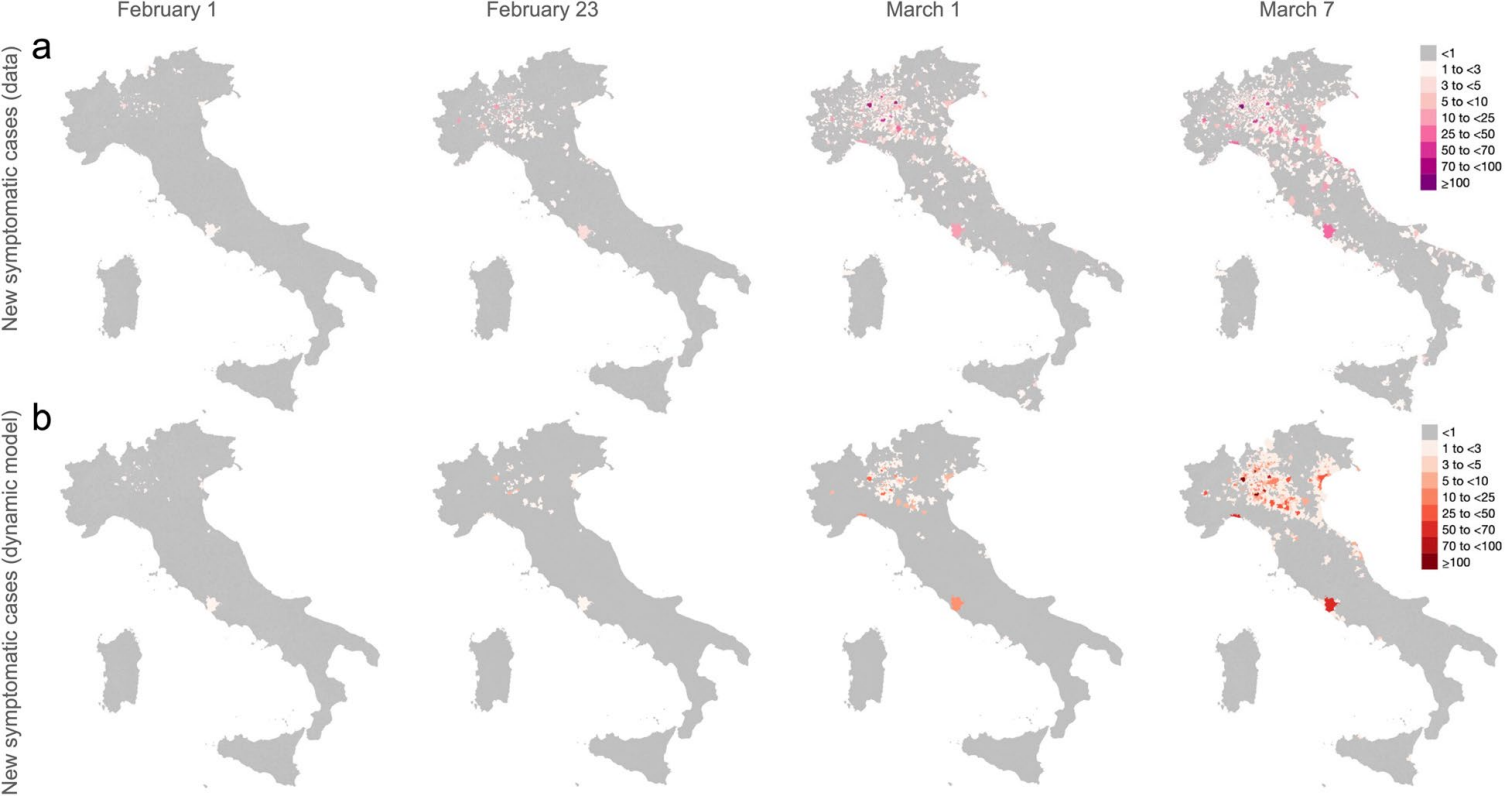
**a** Spatial spread of COVID-19 cases with date of symptom onset from February 1 to March 7, 2020, across different municipalities of Italy as observed in the data [2]. **b** As **a**, but as obtained by simulating a SIR dynamic transmission model, under the assumption that 3% of infected individuals were ascertained by public health authorities. Panel b shows results for the SIR simulation that minimize the root mean square error with respect to the time series of cases retrospectively identified at the regional level. Mean estimates across all different model simulations are shown in Figure S6

We compare the estimates obtained with the probabilistic model to those obtained with a dynamic SIR model, simulating the temporal spread of all SARS-CoV-2 infections across different municipalities of Italy between February 1 and March 7, 2020. The dynamic SIR model is statistically accurate in identifying regions where at least one symptomatic case was notified at different times (Cohen’s kappa coefficient > 0.5; see Table S1). The relatively lower amount of spatial dispersal estimated by the model with respect to what observed in the data (Figs. 2 and S6) might be partially explained by the intrinsic stochasticity of the transmission process and the likely occurrence of repeated importations of infectious cases from abroad, which are not accounted for in the dynamic model. Nonetheless, a particularly good agreement with data on the number of cumulative cases with symptom onset between February 1 and March 7, 2020, is found for regions with more than 50 daily cases ascertained on March 8 (Figure S7). As an overall measure of goodness of fit, we compute the percentage of variance in the data explained by model estimates at a regional grain (20 regions), which results in a coefficient of determination R^2^ of 0.97 when considering the initial conditions best reproducing the observed time series of cases (see Figures S8 and S9 for more details).

Estimates of the overall cumulative distribution of the geographic distances of transmission occurred up to March 7, 2020 (Fig. 1b) as obtained with the dynamic SIR values well compare with what is evaluated through the probabilistic model. To test the robustness of our results with respect to different values of the ascertainment ratio, we repeat the analysis based on the dynamic SIR model by assuming a 10% case ascertainment ratio up to March 8, 2020. Obtained results were almost identical to those obtained under our baseline assumption (see Figures S10 and S11).

**Fig. 3:**
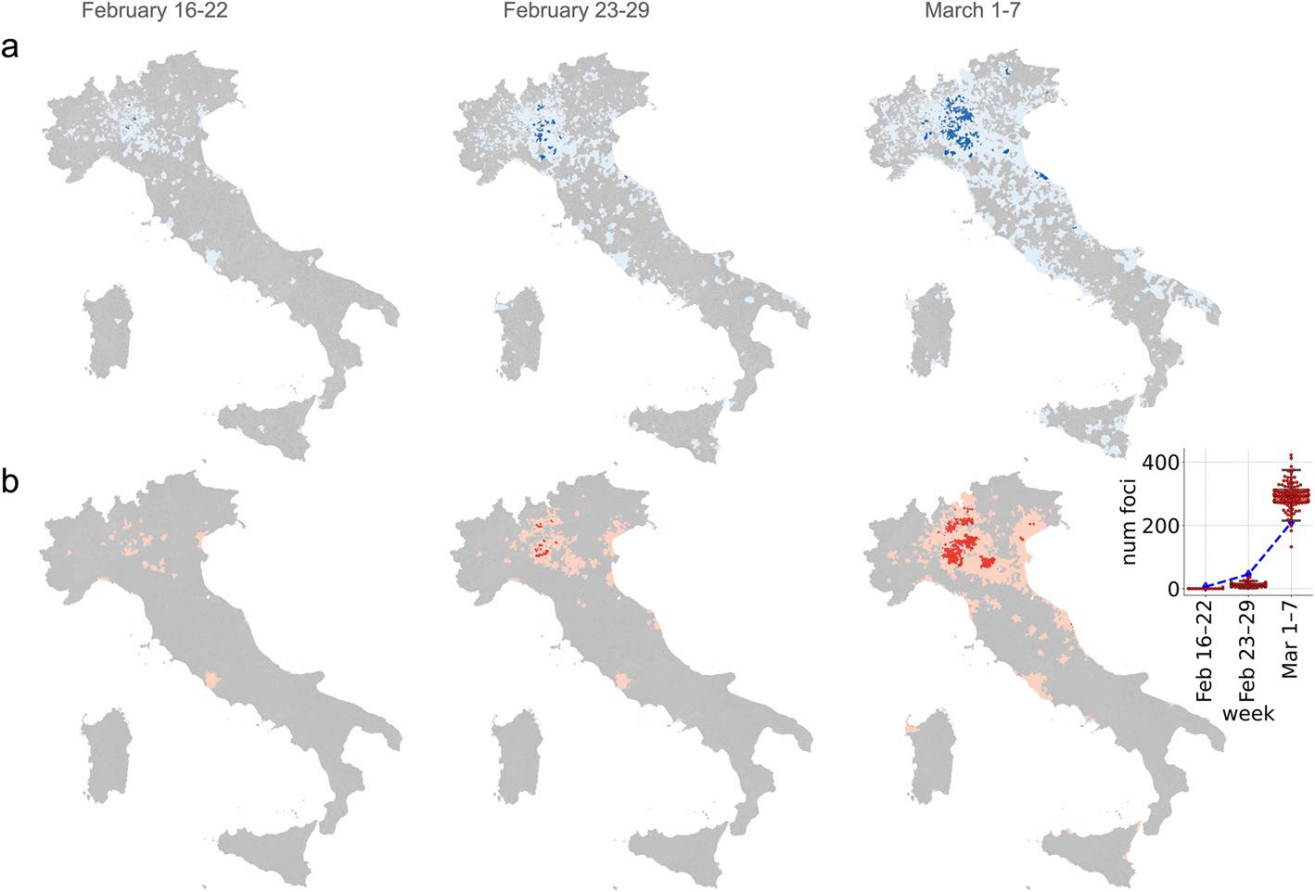
**a** Spatial distributions of potential transmission foci (dark blue) as estimated with the probabilistic approach over 3 different weeks, namely February 16 – 22, February 23 – 29, and March 1 – 7, 2020. Municipalities with at least one individual developing symptoms in the corresponding week are shown in light blue. **b** As **a**, but as obtained by simulating a SIR dynamic transmission model: foci in red, municipalities with at least one notified case in pink. The inset shows the number of epidemic foci as estimated with the probabilistic approach (blue line) and as estimated with 100 simulation runs of the dynamic transmission model (red boxplots)

Using the probabilistic approach, we analyze the progressive increase of potential foci of SARS-CoV-2 transmission in Italy up to March 7, 2020, by identifying for each week those municipalities with 1) more than 10 ascertained symptomatic cases per week, 2) an incidence of at least 1 symptomatic case per 1000 residents per week, and 3) more than 50% of transmission caused by social interactions likely occurring between individuals of the same municipality. According to this definition, only six municipalities are identified as possible transmission foci between February 16 and February 22, 2020. This number increases to 45 between February 23 and February 29, 2020, and to 209 in the first week of March 2020 (Fig. 3a). Consistent results are also obtained when estimating the progressive increase in the number of transmission foci in Italy through the dynamic SIR model. Specifically, according to simulated epidemic trajectory minimizing the root mean square error with regional data, we estimate 0 (mean across all the considered initial conditions: 0, 95%PI: 0–1), 24 (mean: 12, 95%PI: 3–25), and 257 (mean: 293, 95%PI: 206–381) foci for the weeks February 16 – 22, February 23 – 29, and March 1 – 7, respectively (Fig. 3b).

### Impact of local restrictions based on ascertained cases on the spatial spread of SARS-CoV-2

**Fig. 4:**
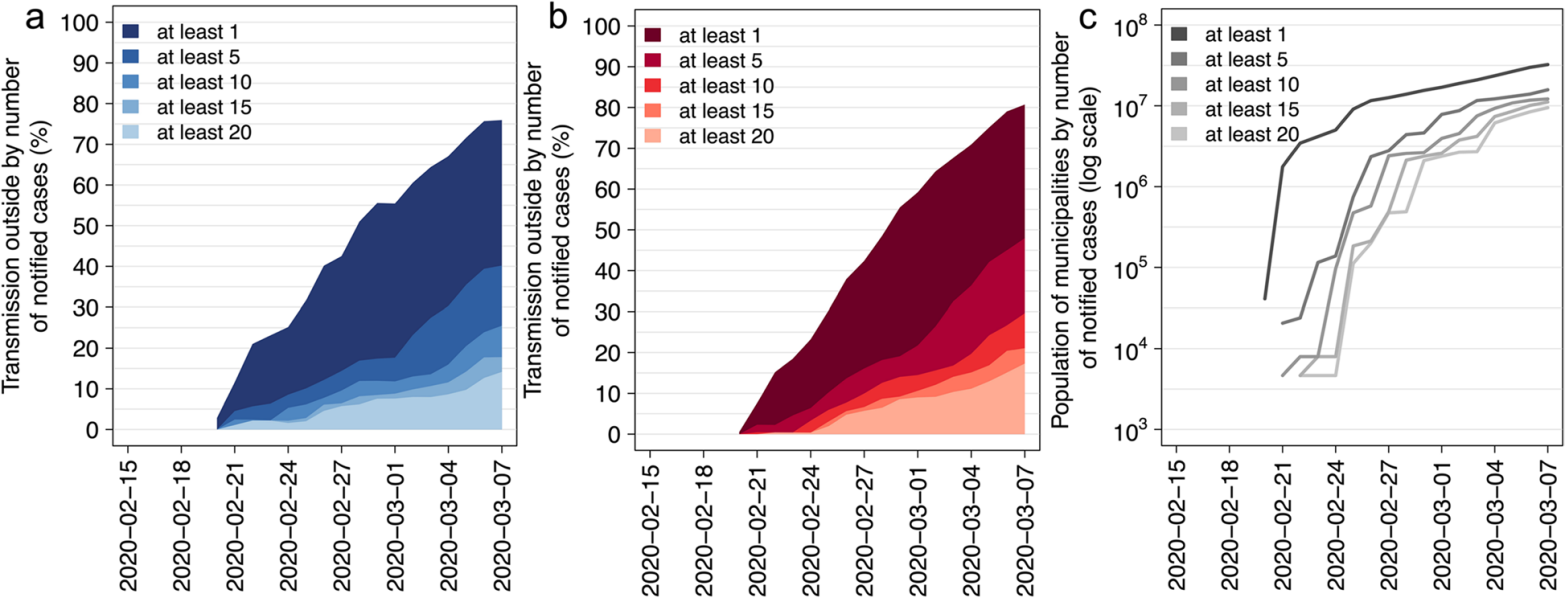
**a** Percentage of transmission ascribable to infected individuals residing in municipalities with at least 1, 5, 15, 20 cumulative notified cases in the data, as estimated with the probabilistic approach at different times. **b** As **a**, but as obtained by simulating a SIR dynamic transmission model. **c** Total number of individuals residing in municipalities with at least 1, 5, 15, 20 cumulative notified cases in the data

Despite our results suggest that, on February 23, most of the cases were infected at short distances (≤ 10 km) from their municipality of residence, municipalities with at least 10 cumulative notified cases in the data accounted only for less than 5% of the inter-municipality spillover of the infection over the national territory, representing transmission episodes occurring because of contacts between individuals residing in different municipalities (Fig. 4). This result highlights that, at that time, prohibiting residing individuals to exit these municipalities would have had a marginal impact on the spatial spread of SARS-CoV-2. All the municipalities with at least 1 ascertained symptomatic or asymptomatic infection (counting more than 3 million citizens in total), were likely responsible for only 20% of spillover episodes at the national level. On March 7, 2020, the day before a lockdown was imposed in the entire Lombardy Region, a policy to limit the mobility outside the municipality of residence for individuals residing in those municipalities with at least one notified positive individual would have left more than 20% of spillover transmission uncontrolled, while affecting almost 32 million Italian citizens (~ 54% of the total population). The same policy imposed only on municipalities with at least ten ascertained cases would have left uncontrolled more than 75% of spillover transmission and would have affected about 10 million individuals (~ 17% of the total population).

Comparable results are obtained with the two considered modeling approaches (probabilistic model vs dynamic SIR model; see Fig. 4).

## Discussion

Estimates on the probability of observing transmission episodes between individuals at different distances are instrumental for defining appropriate strategies to control the spread of infectious diseases. The timely identification of areas representing the main source of infection and contributing the most to the disease spread is a key factor to settle effective containment and control measures.

We investigate the spatiotemporal dynamics of SARS-CoV-2 in Italy before the first European lockdown was imposed in Lombardy region on March 8, 2020. To do this, we use a probabilistic model informed with symptomatic cases that occurred over time in each municipality of the country as identified by contact-tracing operations and retrospective epidemiological investigations of ascertained infections.

Our analysis provides estimates of the probability of transmission at different distances, showing when the infection dynamics switched from the phase when most of the transmission occurred because of the erratic spillover and importation of cases from most affected areas to a phase where the spread of the infection was locally sustained. Obtained results also give insights into the spatial distribution of transmission foci in Italy at the very early stages of the pandemic. We also highlighted the potential effect of quarantining only those municipalities where COVID-19 cases were identified in terms of the percentage of averted spillover transmission from affected areas into the rest of the country. We find that the probability of transmission at short distances progressively increased during the initial pandemic phase, with the proportion of short-distance transmission (≤ 10 km) rising from less than 40% in early February to more than 80% in the first week of March 2020. These findings suggest that at the beginning of March, when major restrictions on physical distancing were imposed to the general population in Italy, the spread of COVID-19 was predominantly driven by contacts occurring within each single municipality or through short-distance travels. At this date, we estimate that more than 200 transmission foci were contributing to the spread of SARS-CoV-2 over the Italian territory, but that less than 25% of the spillover transmission episodes occurring inter municipalities were ascribable to municipalities with more than 10 notified cases. On March 7, 2020, the day before a lockdown was imposed on all residents of the Lombardy Region, municipalities with no ascertained infections were likely contributing to more than 20% of spatial spread of SARS-COV-2 in Italy.

The analysis of detailed spatiotemporal records of COVID-19 symptomatic cases occurred in Italy in early 2020 therefore suggests that—shortly after the first autochthonous case was diagnosed in the country—policies focusing on reducing the transmission of SARS-CoV-2 within each municipality and applied at national level were required to interrupt the disease spread. In fact, given the estimated large proportion of short-distance transmission and the large number of epidemic foci over the national territory, restrictions applied only to individuals residing in municipalities with ascertained cases or limiting only the inter-municipality mobility would have not been sufficient to counter the surge of COVID-19 cases.

Combined with evidence from previous studies [27, 28], with the relatively low SARS-CoV-2 infection ascertainment ratio [26], and with the possibly low detection rates of cases imported from abroad [29], our analysis highlights the need for an immediate response based on multiple non-pharmaceutical interventions (encompassing among others bans of mass gatherings, school closures, and stay-at-home orders) applied at national level during the early phase of the COVID-19 pandemic.

The following limitations should be considered when interpreting the presented results. Local restrictions applied before March 7, 2020, as well as measures applied at the national level shortly after the first diagnosis of SARS-CoV-2 on February 23, were not investigated in the proposed analysis. In addition, the number of cases ascertained in low-transmission settings is subject to inherent biases in clinical case reporting, and our results could be affected by the potential heterogeneity across Italian regions in the ascertainment ratio of SARS-CoV-2 infections during the early pandemic phase. Changes in the reporting that occurred in the early stage of the pandemic cannot be excluded as well.

In our analysis, the use of a radiation model allowed us to keep as simple as possible the human mobility framework, while overcoming potential biases associated with mobility patterns inferred from empirical data representing only specific periods or age-segments of the population. Although mobility patterns are expected to dramatically change as a consequence of interventions, it has been proven that in the early stages of an epidemic, the use of real data on individuals’ movements does not increase forecast performance compared to a non-parametric radiation model [30]. We showed that the radiation model is reasonably able to capture available data on individuals’ commuting (Figures S2 and S3) as well as the overall temporal increase of COVID-19 cases across regions (Figures S7-S9). Nonetheless, the proposed deterministic dynamic SIR model is not appropriate to describe the exact trajectory and spatial dispersal of SARS-CoV-2 infections identified during the initial pandemic phase. While alternative modeling approaches would better reflect the stochastic nature of the transmission process and the disease spread in the population, the development of such models would require additional assumptions on the epidemic seeding, considering repeated importation of cases from abroad. Finally, the spatial resolution of the analyzed data was insufficient to disentangle the contribution of within-household transmission to the disease spread.

Despite these limitations, our study represents a first attempt to quantify the probability of transmitting the SARS-CoV-2 infection at different distances in the absence of strict restrictions, and the contribution of the municipalities where the transmission was already detected to the initial spread of COVID-19 in Italy.

### Supplementary Information

**Supplementary Material 1.**

## Data Availability

The data contain confidential information, and public data deposition is not permitted. Due to the sensitive nature of the data, data can only be made available by the Istituto Superiore di Sanità (Italian National Institute of Health) through a data-sharing agreement directly with the user (contact mail: patrizio.pezzotti@iss.it).
