# Supplementary material for: Spatial spread of COVID-19 during the early pandemic phase in Italy
Source: BMC Infect Dis. 2024 Apr 29;24:450. doi: 10.1186/s12879-024-09343-8 (PMC11057115; doi:10.1186/s12879-024-09343-8)
Supplement: Supplementary file 1 — Supplementary Material 1. [file 12879_2024_9343_MOESM1_ESM.docx]

**Supplementary Material for:**

**Spatial spread of COVID-19 during the early pandemic phase in Italy**

Valeria d’Andrea^a,b,#^, Filippo Trentini^a,c,d,#^, Valentina Marziano^a^, Agnese Zardini^a^, Mattia Manica^a^, Giorgio Guzzetta^a^, Marco Ajelli^e^, Daniele Petrone^f,g^, Martina Del Manso^f^, Chiara Sacco^f^, Xanthi Andrianou^f^, Antonino Bella^f^, Flavia Riccardo^f^, Patrizio Pezzotti^f,§^, Piero Poletti^a,§^, and Stefano Merler^a,§,*^.

1. Center for Health Emergencies, Fondazione Bruno Kessler, Trento, Italy
2. Department of Physics and Astronomy “Galileo Galilei”, University of Padua, Padua, Italy
3. Dondena Centre for Research on Social Dynamics and Public Policy, Bocconi University, Milan, Italy
4. Department of Decision Sciences, Bocconi University, Milan, Italy
5. Laboratory for Computational Epidemiology and Public Health, Department of Epidemiology and Biostatistics, Indiana University School of Public Health, Bloomington, IN, USA
6. Department of Infectious Diseases, Istituto Superiore di Sanità, Rome, Italy
7. Department of Statistics, Sapienza University of Rome, Rome, Italy

^#^ joint first authors

^§^ joint senior authors

^*^ Corresponding author: merler@fbk.eu

**This file includes:**

Figures S1 to S11


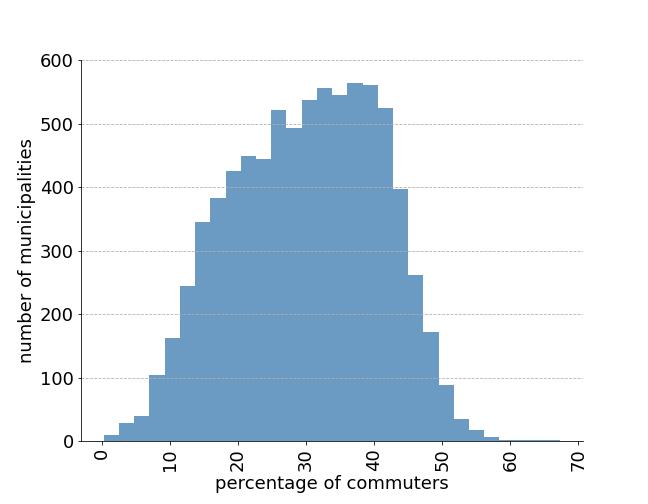


**Figure S1.** Distribution of the resident population in each municipality that daily commutes to other municipalities, according to the Italian National Institute of Statistic(1).


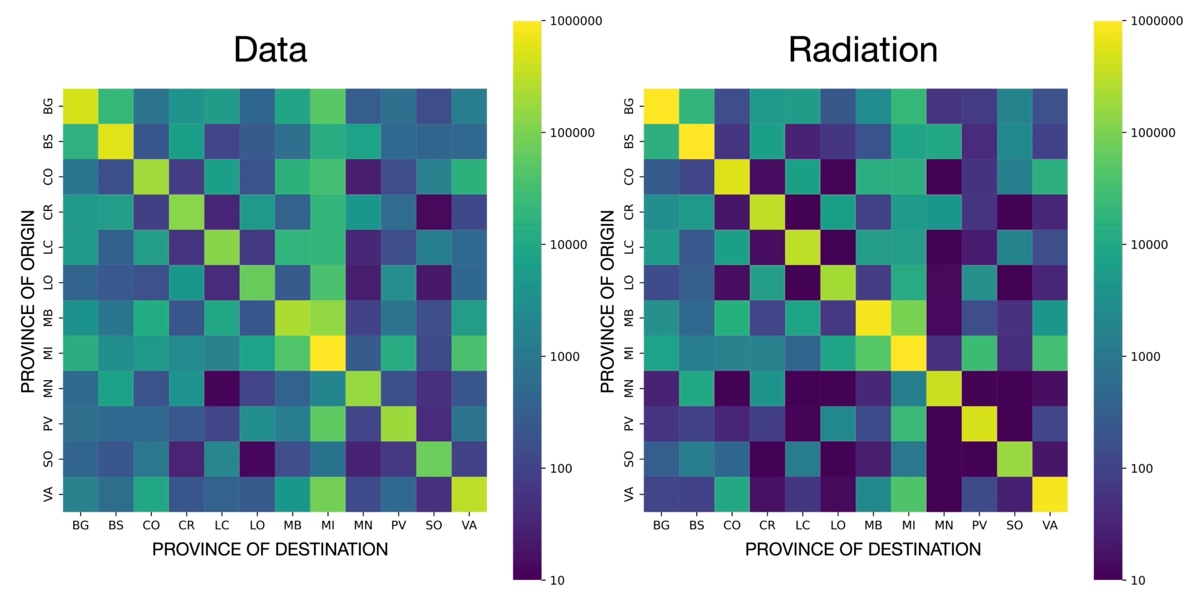


**Figure S2.** Comparison between the mobility fluxes (number of travelers) among the 12 provinces of Lombardy region estimated according to the projection for year 2020 made by Lombardy region based on 2016 census data (2) (right) and those estimated with the radiation model (right).


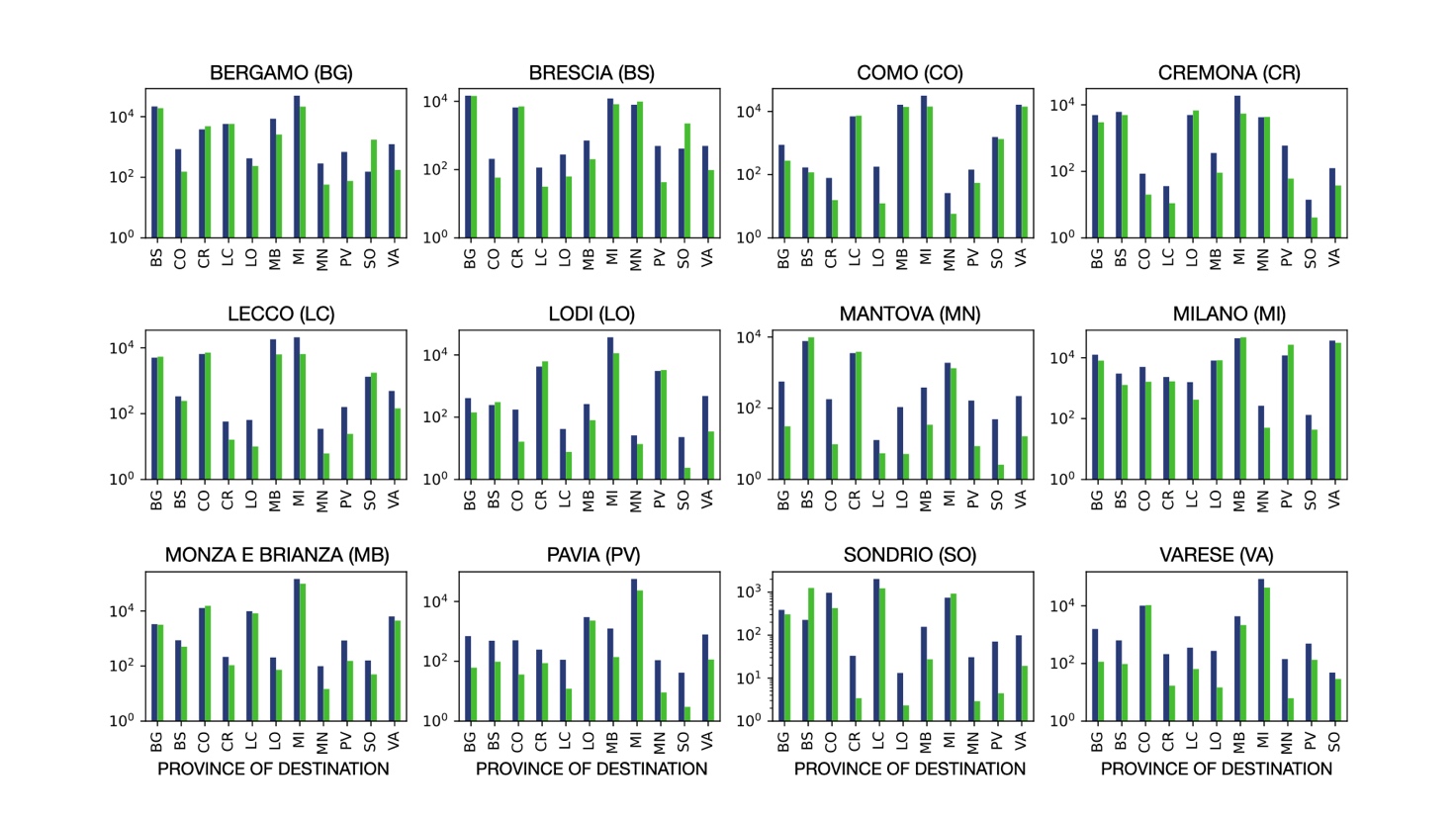


**Figure S3.** Comparison between the outgoing mobility patterns (fraction of people commuting from one province, top labels, to all others, horizontal axis label) among the 12 provinces of Lombardy region according to the projection for year 2020 made by Lombardy region based on 2016 data (2) (blue bars) and according to the radiation model (green bars).

**Figure S4.** **a** Daily number of notified COVID-19 symptomatic cases in Italy and number of municipalities with at least one new notified case, by date of diagnosis, 26 January–08 March 2020. **b** Daily number of new COVID-19 symptomatic cases in Italy and number of municipalities with at least one new symptomatic case, by date of symptoms onset, 26 January–08 March 2020, as retrospectively consolidated through epidemiological investigations.


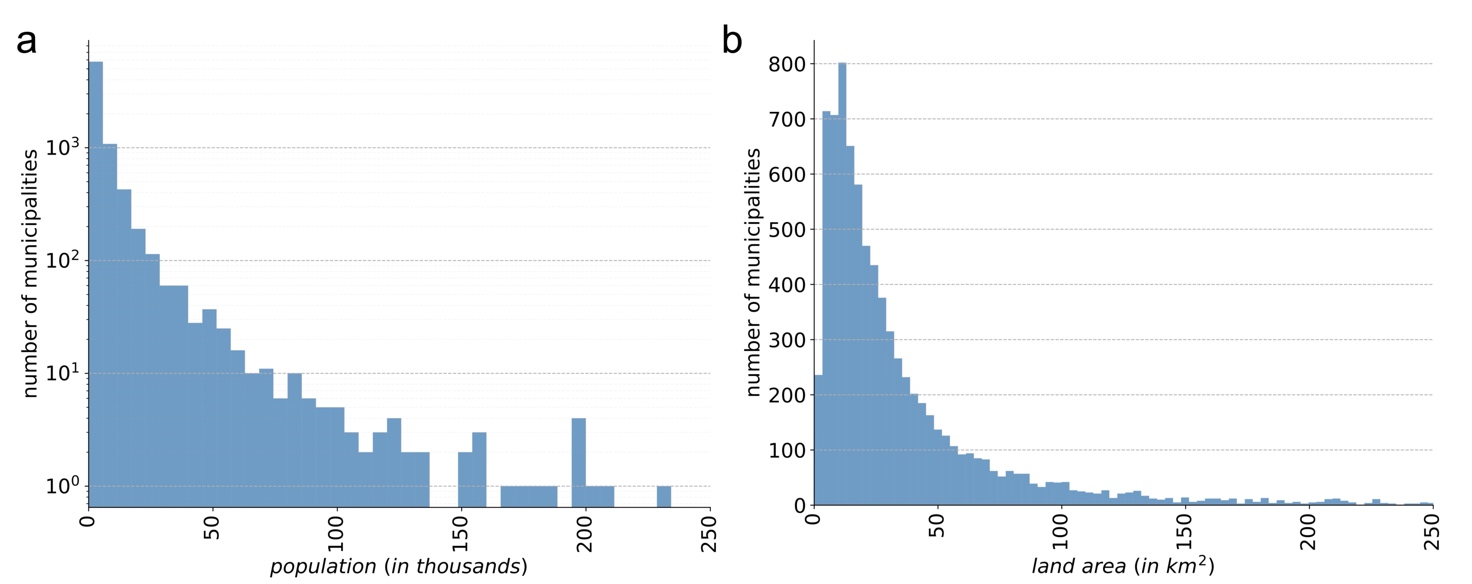


**Figure S5. a** Distribution of the population size (in thousands of individuals) residing across the 7926 municipalities of Italy, according to the Italian National Institute of Statistics(1). **b** Distribution of the land area (in km2) covered by the 7926 municipalities of Italy, according to the Italian National Institute of Statistics. The latter shows that 95% of Italian municipalities have a land area between 4 and 123 km^2^ (median: 22).


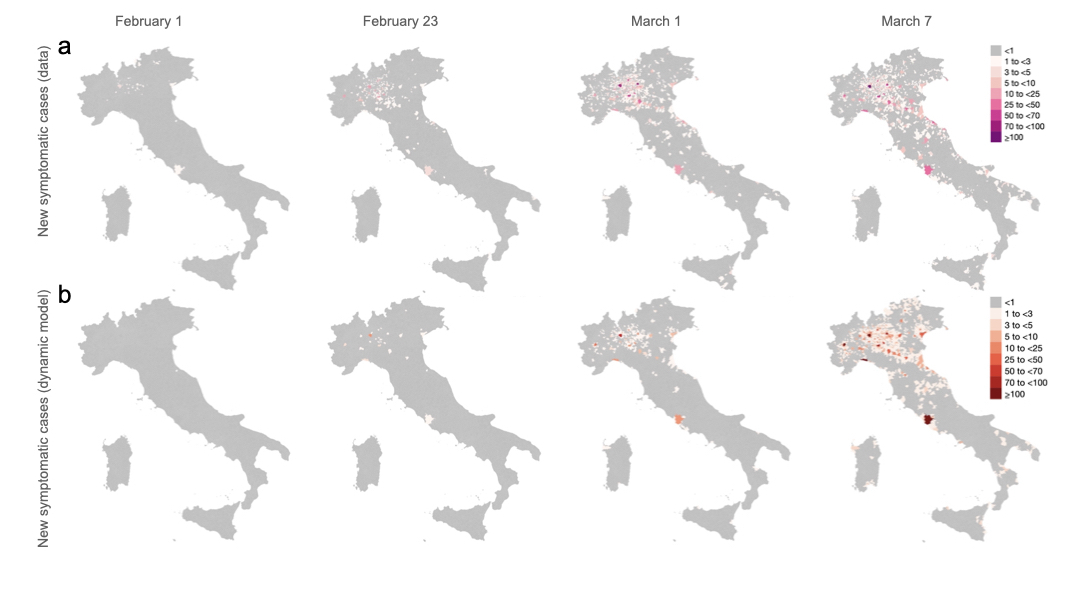
**Figure S6. a** Spatial spread of COVID-19 cases with date of symptom onset from February 1 to March 7, 2020, across different municipalities of Italy as observed in the data. **b** As **a**, but as obtained by averaging the model estimates obtained with 100 different initial conditions for the SIR dynamic transmission model, under the assumption that 3% of infected individuals were ascertained by public health authorities.

**
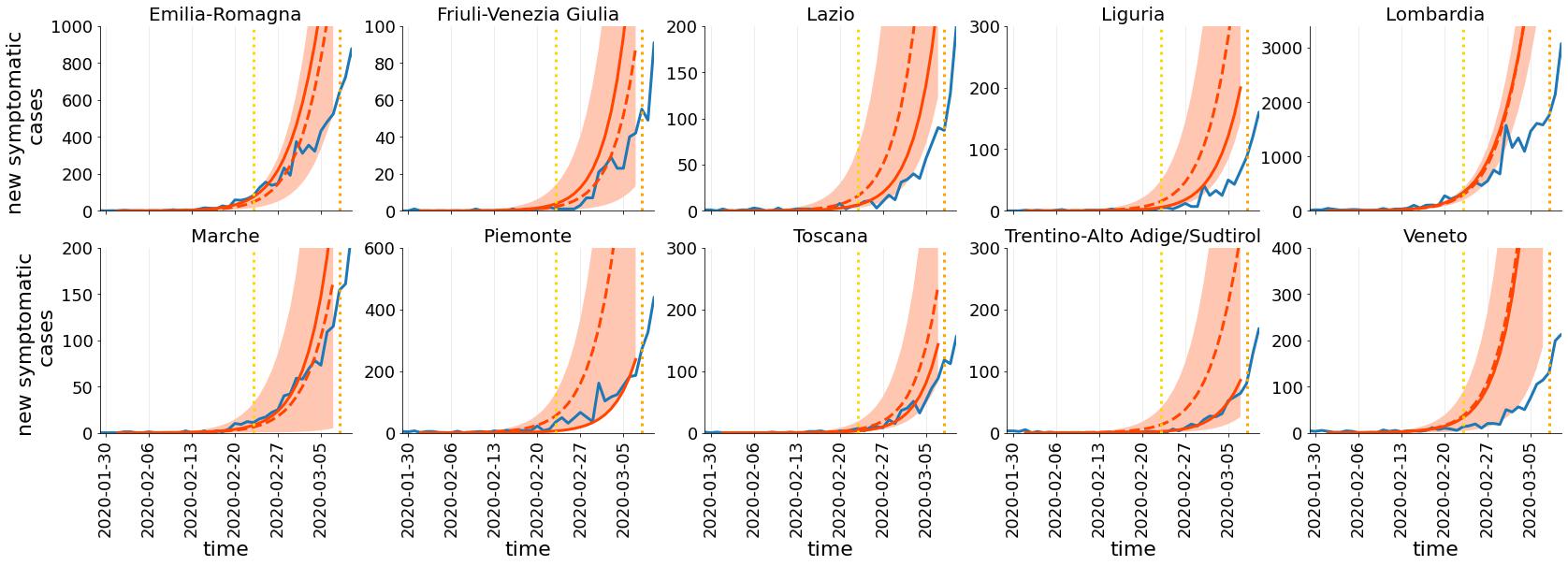
**

**Figure S7.** Temporal dynamics of new symptomatic individuals in regions with more than 50 daily new symptomatic cases on March 8. Data (blue line), model mean (red dotted line), 95%PI (red shaded area), and the SIR realization, among the 100 simulated, whose initialization on February 1st, 2020, led to an epidemic trajectory that best explains the pattern of cases retrospectively identified at the regional level up to March 7th, 2020 (red solid line). The best seeding identified is the one associated with large values of R^2^ (0.97%) and of Cohen’s kappa coefficient (see Figure S9 and Table S1). Vertical dashed lines correspond to February 23 (yellow line) and to March 8 (orange line) 2020, respectively representing the date when first strict physical distancing measures were imposed in 11 municipalities in Lombardy and Veneto and the date when a national lockdown was imposed.


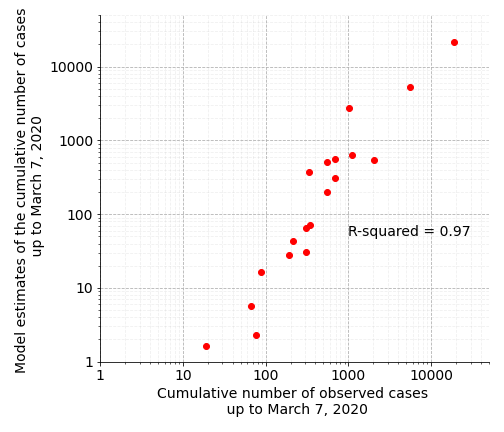


**Figure S8.** Number of cumulative cases with symptom onset between February 1 and March 7, 2020, as retrospectively identified by public health authorities in Italy (x-axis) and as estimated using the dynamic SIR model (y-axis shows the best model realization among explored initial conditions). Different points correspond to the different Italian regions.


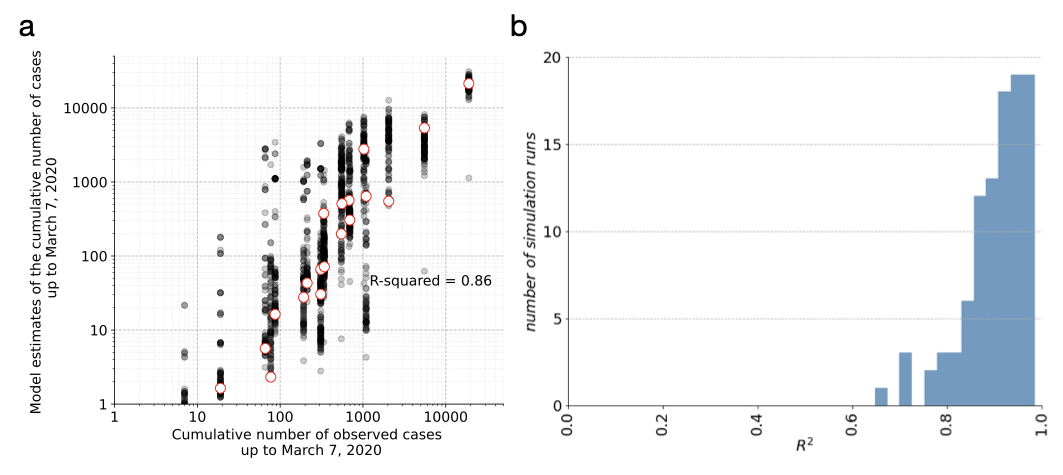


**Figure S9. a** Number of cumulative cases with symptom onset between February 1 and March 7, 2020, as retrospectively identified by public health authorities in Italy (x-axis) and as estimated using the dynamic SIR model (y-axis); grey dots refer to different epidemic seeding considered with the model; empty red dots correspond to the best epidemic trajectory across different initial conditions explored with this model. **b** Distribution of coefficients of determination R^2^ computed as the percentage of variance in the data (that is the number of cumulative cases with symptom onset between February 1 and March 7, 2020, identified by public health authorities at a regional level) explained by each of the different SIR simulated trajectories, corresponding to different initial conditions of the model.


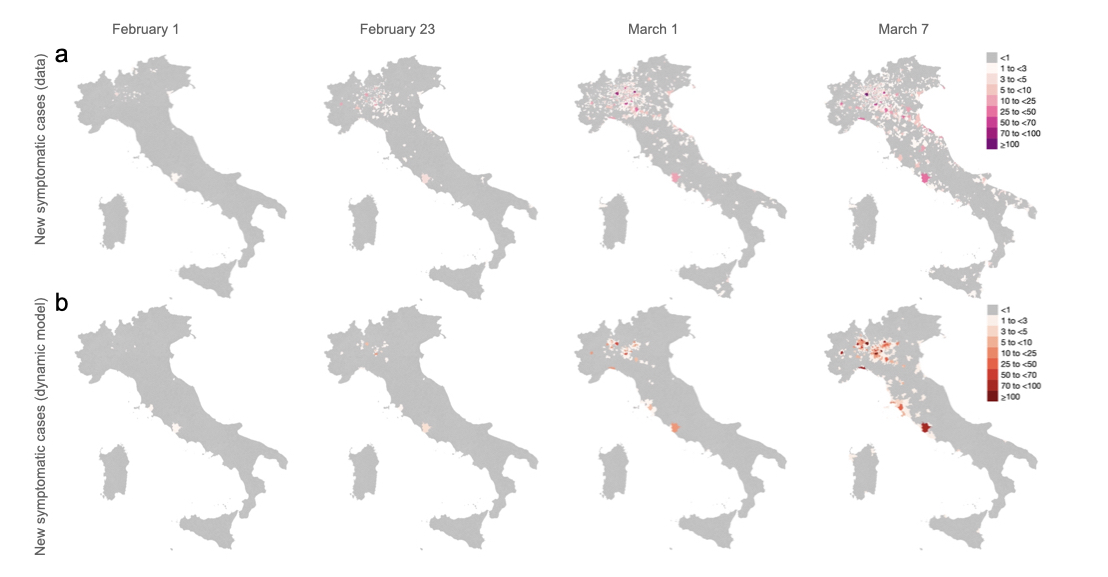


**Figure S10. a** Spatial spread of COVID-19 cases with date of symptom onset from February 1 to March 7, 2020, across different municipalities of Italy as observed in the data (2). **b** As **a**, but as obtained by simulating a SIR dynamic transmission model, under the assumption that 10% of infected individuals were ascertained by public health authorities. Results show that, by assuming an infection ascertainment ratio of 10%, the model estimates an initially lower spatial dispersal of symptomatic cases compared to the baseline assumption (ascertainment ratio: 3%) and to the observed one.


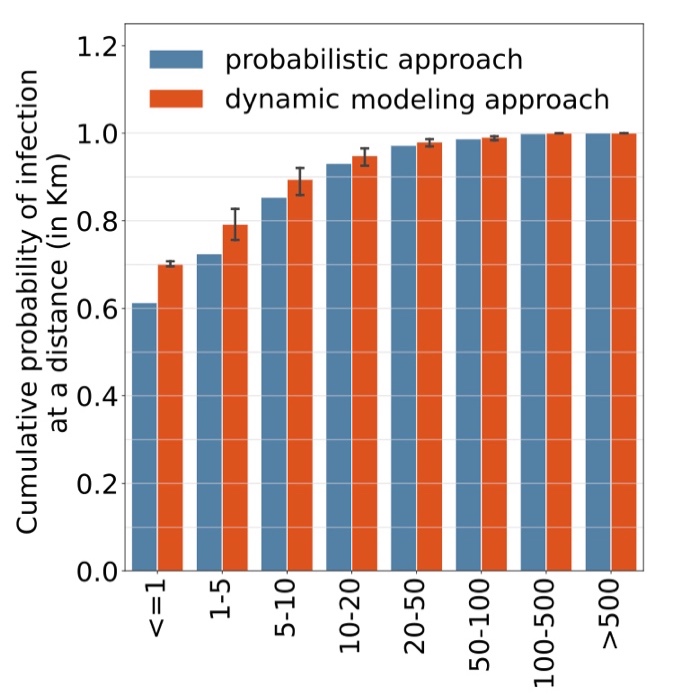


**Figure S11.** Cumulative distribution of the probability that a COVID-19 case was infected at a distance 𝐷 from their residence, as estimated with the probabilistic approach (blue) and the dynamic SIR model (red) under the assumption of 10% of infected individuals were ascertained by public health authorities. The considered time interval spans between January 26 and March 7, 2020. Vertical lines show the range from 2.5 to 97.5 percentiles associated with 100 simulation runs.

| Time interval | Cohen’s k  Mean (95% PI) | Cohen’s k  Best epidemic trajectory |
| --- | --- | --- |
| Feb 01-Feb 23 | 0.42 (0.40- 0.45) | 0.50 |
| Feb 01-Mar 01 | 0.54 (0.51-0.58) | 0.67 |
| Feb 01-Mar 07 | 0.66 (0.62-0.70) | 0.77 |

**Table S1.** Cohen's kappa coefficient evaluated at the regional level for different time intervals by comparing regions with at least one notified case in the data with the corresponding estimates obtained with a dynamic deterministic SIR model. Performances shown for the best epidemic trajectory refer to those obtained with the SIR simulation that minimize the root mean square error with respect to the time series of cases retrospectively identified at the regional level.

1. Istituto Nazionale di Statistica. Informazioni territoriali e cartografiche [Internet]. Available from: https://www.istat.it/it/informazioni-territoriali-e-cartografiche

2. Regione Lombardia. Matrice OD2020 - Passeggeri [Internet]. Available from: https://www.dati.lombardia.it/Mobilit-e-trasporti/Matrice-OD2020-Passeggeri/hyqr-mpe2/about_data
